# Distribution Committee's Report

**Published:** 1907-08-10

**Authors:** 


### DISTRIBUTION COMMITTEE'S REPORT.

The Committee of Distribution held their first meeting on Tuesday, May 23, and at that and subsequent meetings examined the reports and income and expenditure accounts of the several institutions seeking to participate, and made their awards in conformity with Law V.

The executors of the late Mr. George Herring will pay to the treasurer of this fund on August 1 £30,000, on account of the legacy bequeathed to the Hospital Sunday Fund, being a sum about equivalent to one year's interest which Mr. Herring would have received on the amount of the residuary bequest.

The committee recommend that this amount be added to the collection, making the total amount of the fund up to the present time £74,165, in which is included a second payment of £3,000 on account of the legacy of £10,000 under the will of the late Mr. Herbert Lloyd. This year 252 institutions have made applications for grants from the fund, being two more than in 1906. Seven hospitals and two nursing associations which applied for grants were considered ineligible. The committee, though fully realising the excellent work carried on by the Cancer Hospital, have not made an award this year, for the reason stated in their report of last year ["the hospital not being in want of funds at the present time"]. The committee now recommend the distribution of £68,134 3s. 4d. to the 159 hospitals and institutions, 57 dispensaries, and 27 nursing associations shown in the appendix attached to the report. The committee having reduced the bases of award in 29 cases, this fact was intimated to each hospital interested in accordance with Law V. Eight deputations attended the committee, and after hearing their cases the final awards were determined. Five per cent. of the total sum available for distribution is set apart to purchase surgical appliances during the ensuing year.

